# Localized interleukin-12 delivery for immunotherapy of solid tumours

**DOI:** 10.1111/jcmm.12121

**Published:** 2013-11-19

**Authors:** Louis Z Wei, Yixin Xu, Megan E Nelles, Caren Furlonger, James CM Wang, Marco A Di Grappa, Rama Khokha, Jeffrey A Medin, Christopher J Paige

**Affiliations:** a Department of Immunology, University of TorontoToronto, ON, Canada; b Ontario Cancer Institute, University Health NetworkToronto, ON, Canada; c The State Key Laboratory of Pharmaceutical Biotechnology Division of Immunology Medical School, Nanjing UniversityNanjing, China; d Department of Medical Biophysics, University of TorontoToronto, ON, Canada; e Institute of Medical Sciences, University of TorontoToronto, ON, Canada

**Keywords:** Interleukin-12, immunotherapy, solid tumour, lentivirus, gene therapy

## Abstract

Interleukin (IL)-12 is the key cytokine in the initiation of a Th1 response and has shown promise as an anti-cancer agent; however, clinical trials involving IL-12 have been unsuccessful due to toxic side-effects. To address this issue, lentiviral vectors were used to transduce tumour cell lines that were injected as an autologous tumour cell vaccine. The focus of the current study was to test the efficacy of this approach in a solid tumour model. SCCVII cells that were transduced to produce IL-12 at different concentrations were then isolated. Subcutaneous injection of parental SCCVII cells results in tumour development, while a mixture of IL-12-producing and non-producing cells results in tumour clearance. Interestingly, when comparing mice injected a mixture of SCCVII and either high IL-12-producing tumour cells or low IL-12-producing tumour cells, we observed that mixtures containing small amounts of high producing cells lead to tumour clearance, whereas mixtures containing large amounts of low producing cells fail to elicit protection, despite the production of equal amounts of total IL-12 in both mixtures. Furthermore, immunizing mice with IL-12-producing cells leads to the establishment of both local and systemic immunity against challenge with SCCVII. Using depletion antibodies, it was shown that both CD4^+^ and CD8^+^ cells are crucial for therapy. Lastly, we have established cell clones of other solid tumour cell lines (RM-1, LLC1 and moto1.1) that produce IL-12. Our results show that the delivery of IL-12 by cancer cells is an effective route for immune activation.

## Introduction

The immune system is capable of recognizing and eliminating cancer cells, but fails to do so completely when cancers arise. In fact, tumour-specific cytotoxic T cells (CTL) and tumour antigen-specific antibodies are frequently present even as tumour cells increase in number and metastasize. In these cases, the tumours are able to either decrease their immunogenicity, avoiding detection, or actively suppress the immune system. Indeed, there have been many reports that tumour cells exploit the microenvironment to induce a tolerogenic environment enabling them to thrive 1. For instance, many tumours express interleukin-10 (IL-10). Interleukin-10 has been shown to suppress the function of antigen-presenting cells by reducing their expression of major histocompatibility complex (MHC) and co-stimulatory molecules. This leads to an environment that suppresses Th1 function and promotes T-regulatory cell responses 1.

Immunotherapy for cancer seeks to activate or redirect the immune response against tumours. One of the strategies to achieve this is based on induction of immunomodulatory cytokine expression. Cytokines are important in shaping both the innate and adaptive immune responses, and they have been found to exert key effects on tumour biology 2. Cytokines that are involved in activation of immune effector mechanisms include Type I IFNs 3, IL-2 and granulocyte macrophage colony-stimulating factor (GM-CSF) 4–5, making them excellent candidates for immunotherapy. Another cytokine that has great potential for immunotherapy is IL-12.

Interleukin-12 is a heterodimeric protein made of p35 and p40 subunits, which are joined by a disulphide bond to form the bioactive IL-12p70 protein 6. As one of the first cytokines to be produced in the presence of an antigen, it plays a key role in priming Th1 responses 7. Interleukin-12 is secreted exclusively by antigen-presenting cells such as dendritic cells (DC), monocytes, and macrophages 7, and it plays an important role in activation and proliferation of NK and T cells 7–8. Delivery of recombinant IL-12 (rIL-12) has shown significant stimulatory effects in pre-clinical models, resulting in delayed tumour onset or tumour rejection 9,10. Unfortunately, systemic injection of rIL-12 as a therapy in early human clinical trials showed few objective responses and severe toxicity in some patients 12. Despite this, the well-documented biological effects of IL-12 indicate that it has potential for being a very effective therapeutic compound, prompting investigation into new methods of IL-12 delivery designed to avoid unacceptable toxicity.

Among the various methods envisioned to deliver this potent immune-stimulatory molecule, local administration of IL-12 at the site of the tumour has attracted much attention 13–18. Local injections of adenovirus vectors carrying the IL-12 transgene have been shown to control and suppress established prostate cancer in murine models 15. Along similar lines, tumour-specific CD8^+^ T cells engineered to produce IL-12 resulted in superior therapeutic effects compared with direct injection of rIL-12 16.

We previously published results obtained from experimental murine models that leukaemia cells transduced with a lentiviral vector engineered to express IL-12 are effective immunizing agents 11. Mice not only reject the initial IL-12-producing leukaemia cells but also reject non-transduced, non-IL-12-producing leukaemia cells. The immune response we observed was specific to the leukaemia cell line used for immunization as other established leukaemia lines readily progressed after injection. We also found that only one in 200 leukaemia cells needed to be secreting IL-12 to achieve complete protection as long as those particular cells attained a certain threshold of IL-12 production 11. The immune response was thus curative, specific, and long-lasting.

While similar results were obtained using several additional leukaemia cell lines, a critical and unanswered question was whether this approach would be effective against solid tumours, which are far more prevalent in humans. This question was addressed in the present study using the squamous cell carcinoma cell line, SSCVII, a model of head and neck cancer. SCCVII is a tumour cell line isolated from a spontaneous sarcoma that developed in C3H/HeJ mice. When injected subcutaneously (s.c.) into the flank of mice, it rapidly develops a tumour characterized by dense tumour cell growth, low immune infiltration, low catenation and poor immunogenicity 19. When these cells are engineered to express IL-12, we demonstrate that they also lead to potent immune activation, resulting in recognition and elimination of not only the IL-12-producing sarcoma cells used to generate the vaccine but also non-transduced SCCVII cells that do not secrete IL-12. Importantly, similar observations were also found with three additional solid tumour models of osteosarcoma, prostate cancer and Lewis lung carcinoma, indicating the broad utility of this immunotherapeutic approach.

## Materials and methods

### Lentivirus production

Construction of a lentivector containing a fused form of the mouse IL-12 (mIL-12) cDNA was as described in Labbe *et al*. 11. In summary, mIL-12 (p35:p40) was isolated from Plasmid pORF-mIL-12 (InvivoGen, San Diego, CA, USA) by PCR adding EcoRI and BamHI sites downstream and upstream, respectively, of the cDNA and purified by electrophoresis. This sequence was then subcloned downstream of the Elongation Factor 1-alpha (EF1-α) promoter 20. Recombinant lentiviruses were generated by transfection of 293T cells with pHR-mIL-12, accessory plasmids: pCMV, pMDG and pADV, polyethylenimine (PEI), and sodium chloride. Supernatants were then collected and virus concentrated by ultracentrifugation. Viral titre was determined by transducing naïve 293T cells; titre calculations were performed by intracellular staining of transduced 293T cells with an anti-mIL-12 antibody (BD Biosciences, Mississauga, ON, Canada) and analysis by flow cytometry.

### Tumour cell lines

The murine squamous cell carcinoma cell line, SCCVII, was provided by Dr. Richard P. Hill (Ontario Cancer Institute, University Health Network), and the murine osteosarcoma cell line, moto1.1 [21], was developed in the laboratory of Dr. Rama Khokha (Ontario Cancer Institute, University Health Network). The murine prostate cancer cell line, RM-1 was provided by Dr. Jeffrey Medin (Ontario Cancer Institute, University Health Network). The Lewis lung carcinoma cell line, LLC1, was obtained from American Type Culture Collection. All cell lines were maintained in RPMI 1640 (Invitrogen Life Technologies, Burlington, ON, Canada) with 5% heat-inactivated foetal calf serum, 100 μg/ml Penicillin/streptomycin (Multicell; Wisent Inc, St-Bruno, QC, Canada) or 100 μg/ml kanamycin (Gibco, Grand Island, NY, USA), 10 mM (4-(2-hydroxyethyl)-1-piperazineethanesulfonic acid and 5.5 × 10^−5^ M beta-mercaptoethanol (Sigma-Aldrich, Oakville, ON, Canada) at 37°C with 5% CO_2_.

### Transduction of tumour cells

Tumour cell lines (SCCVII, moto1.1, RM-1, LLC1) were transduced at an estimated multiplicity of infection (MOI) of 2. After transgene expression was determined by flow cytometry, transduced tumour cells were subcloned on Terasaki plates (Sarstedt Inc., Montreal, QC, Canada). In brief, cells were plated on Terasaki plates at a density of 0.3 cells/well. Wells containing single cells were identified by visual inspection. Colonies that expanded from wells containing single cells were isolated and assayed for IL-12 expression. This was performed by plating the clones at 10^6^ cells/ml/4 hrs and subsequently measuring the concentration of IL-12 in the supernatants with a commercially available mIL-12-p70 ELISA kit (BD Biosciences).

### Tumour cell growth kinetics

To determine the growth kinetics of parental SCCVII cells and IL-12-expressing SCCVII clones, cells were stained with carboxyfluorescein succinimidyl ester and cultured at 37°C. Cells were then harvested at 4, 24 and 48 hrs time-points post-staining and CFSE fluorescence was determined by flow cytometry. The doubling time of the cell lines was calculated by determining when the population lost half of its original fluorescence.

### Mice

For each of the four tumour cell lines, syngeneic mouse models were used: female C3H/HeJ mice for SCCVII studies, male C57BL/6J mice for RM-1 and LLC1 studies, and female FVB/NJ mice for moto1.1 studies. Mice were ordered from Jackson Laboratories (Bar Harbor, ME, USA) and housed in specific pathogen-free conditions at the Ontario Cancer Institute. All protocols were approved by the OCI Animal Care Committee.

### *In vivo* tumour experiments

Tumour cells were grown in media as above, collected by low-speed centrifugation, and washed with PBS prior to injection. Cells were injected at a concentration of 2 × 10^5^ (SCCVII), 2 × 10^4^ (RM1), and 1 × 10^6^ (LLC1) cells in 200 μl PBS. Injections were performed s.c. into the flank of the recipient mouse. After injection, mice were monitored daily for tumour development and killed by cervical dislocation when the tumour reached 1.5 cm in any dimension. At this point, the tumours, draining lymph nodes and spleens were harvested for analysis. Tumour volume was calculated using the formula: Tumour volume = 4/3 * length * width^2^ * π.

### T-cell Depletion

Specific antibodies were used to deplete mice of CD4^+^ cells, CD8^+^ cells or both populations. The hybridoma GK1.5 was used against CD4, YTS169 was used against CD8 and HB9419 was used as an isotype control. The hybridomas were obtained from the American Type Culture Collection (ATCC) (Manassas, VA, USA); the protocols for growing the cells and purifying the antibodies were the same as described in Labbe *et al*. 11. The mice were injected IP with 200 μg of depletion antibodies on days −14, −1, 3, 7, 14 and 21. The efficacy of depletion was tested in control experiments prior to use in the experimental models (data not shown). Peripheral blood was collected to assay for the presence of these populations *via* flow cytometry. We found that both GK1.5 and YTS169 injected in this manner achieved >99% depletion of their expected target cells (data not shown).

### Blood cytokine analysis

Mice were bled at regular intervals from their saphenous vein. Approximately 100 μl of blood was collected each time in a serum separator tube (BD Biosciences). Tubes were then spun at 319 g for 10 min. and serum was collected. Serum cytokine levels were determined using a flow cytometry-based mouse inflammatory cytokine bead assay (BD Biosciences); IL-10, IL-6, IL-12, Monocyte chemotactic protein 1 (MCP-1), interferon γ (IFN-γ) and tumour necrosis factor α (TNF-α) levels were measured.

### Histology

Tumour samples were isolated from mice at various stages of development. These were either fixed in paraformaldehyde or frozen in optimal cutting temperature compound for immunohistochemistry. Samples were sent to the pathology laboratory in Toronto General Hospital for staining with haematoxylin and eosin and various lymphocyte markers (see below).

### Flow cytometric analysis of TILs

Tumours were extracted from mice and minced to small pieces measuring approximately 1 mm in diameter. The minced tumour was then placed in a digestion media of RPMI 1640 containing 0.5 mg/ml collagenase IV (Sigma-Aldrich) and 0.025 mg/ml DNase I (Roche, Basel, Switzerland). Digestion was performed at 37°C over 90 min. with vigorous vortexing every 15 min. Tumour digests were then passed through a 70 μm filter to remove large undigested pieces, and the remaining material was Fc blocked with αCD16/32 purified antibody (eBioscience, San Diego, CA, USA). The cells were then stained with antibodies against cell surface proteins. T cells were stained with αCD45, αCD4 and αCD8. DCs were stained with αCD11c, αCD80 and αCD86. Lastly, 7AAD was used as a live/dead stain.

### Statistical analysis

Log-rank (Mantel-Cox) test was used to analyse survival graphs.

## Results

### Generation of IL-12-secreting tumour cells

**Figure 1:**
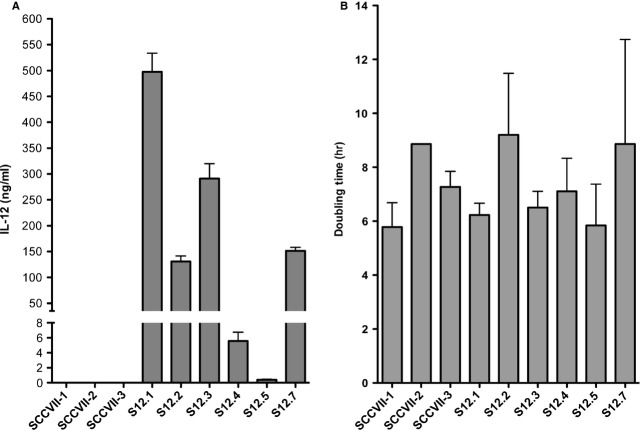
Transduction of SCCVII cells with an interleukin (IL)-12 lentiviral vector led to the isolation of clones that secrete IL-12 at various levels. (A) Secretion of IL-12 by clones when plated at a concentration of 10^6^ cells/ml/4 hrs. (B) doubling time of IL-12-expressing and non-expressing SCCVII clones as calculated by loss of CFSE staining.

The IL-12 lentivector was designed to contain the murine IL-12 p35 and p40 subunits joined by a linker sequence 11. Interleukin-12 expression in transduced cells is driven by an EF1-α promoter, which favours constitutive expression of IL-12 once the recombinant provirus has integrated into the cellular DNA. After transduction with an approximate MOI of 2, single cell clones were isolated using the methods described in materials and methods and assayed for IL-12 production. As seen in Figure 1A, clones (S12.1-7) were isolated that produce IL-12 at different levels (range 0.5–500 ng/ml when cells were at a density of 1 × 10^6^ cells/ml for 4 hrs), three non-transduced SCCVII clones (SCCVII-1-3) were also tested for IL-12 production, all three were below detectable range of the assay. To test if the transduction procedure itself or the production of IL-12 influenced the growth kinetics of the tumour cells, we determined the doubling times of a number of clones. As seen in Figure 1B, when the 6 IL-12-producing clones (S12.1-7) were compared with three non-transduced SCCVII clones (SCCVII-1-3), only modest differences were observed in the doubling time of these cell lines and these did not correlate with IL-12 expression levels.

### IL-12-producing cell lines were able to inhibit tumour development

**Figure 2:**
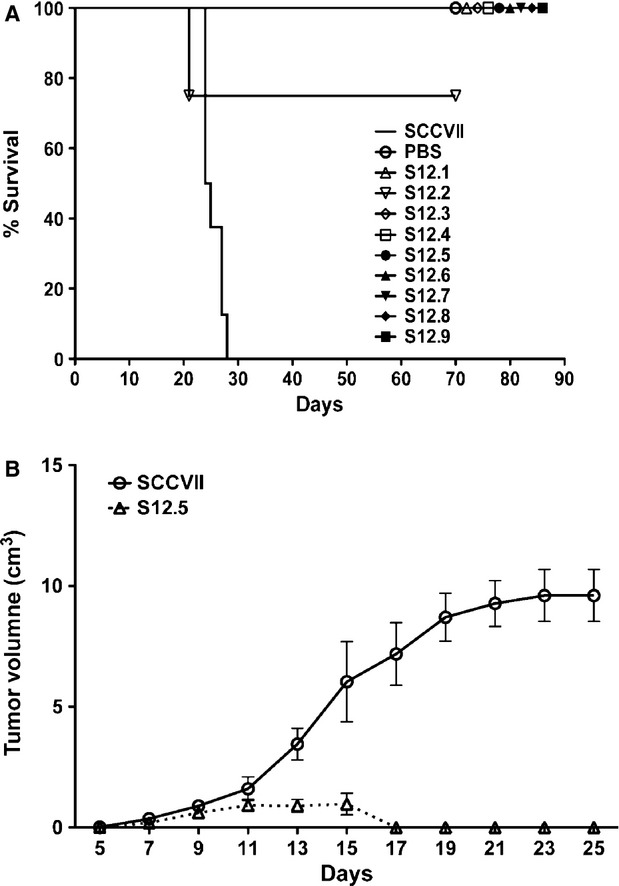
Injection of interleukin (IL)-12-secreting clones protected mice from developing tumours. (A) Either IL-12-secreting clones, S12.1-9, or non-secreting SCCVII were injected at a concentration of 2 × 10^5^ cells/200 μl s.c. into the right flank of C3H/HeJ mice. IL-12-secreting cells protected from tumour development (*P* < 0.05, *n* = 5, log-rank test) compared with SCCVII; PBS injection was used as a control. (B) Tumour growth of a non-secreting SCCVII clone and IL-12-secreting S12.5 when injected s.c. into the right flank of mice. (*P* < 0.05, *n* = 5, log-rank test).

The IL-12-producing SCCVII clones were injected s.c. into the right flank of C3H mice and the animals were monitored for tumour growth. As seen in Figure 2A, all mice injected with the SCCVII parental cell line developed tumours that reached a size of 1.5 cm, at which point, animals were killed following institutional guidelines. In contrast, mice injected with IL-12-producing tumour cells were all protected from tumour development (*P* < 0.05, *n* = 5). Of interest, the SCCVII cell line producing the lowest amount of IL-12 (0.5 ng/ml/10^6^ cells/4 hrs) did generate a noticeable tumour that reached a maximum volume of 1 cm^3^ before receding (Fig. 2B). In all other cases, the IL-12-producing cells were eliminated before any obvious tumours were generated.

### Both CD4^+^ and CD8^+^ cells are required to eliminate IL-12-producing tumour cells

**Figure 3:**
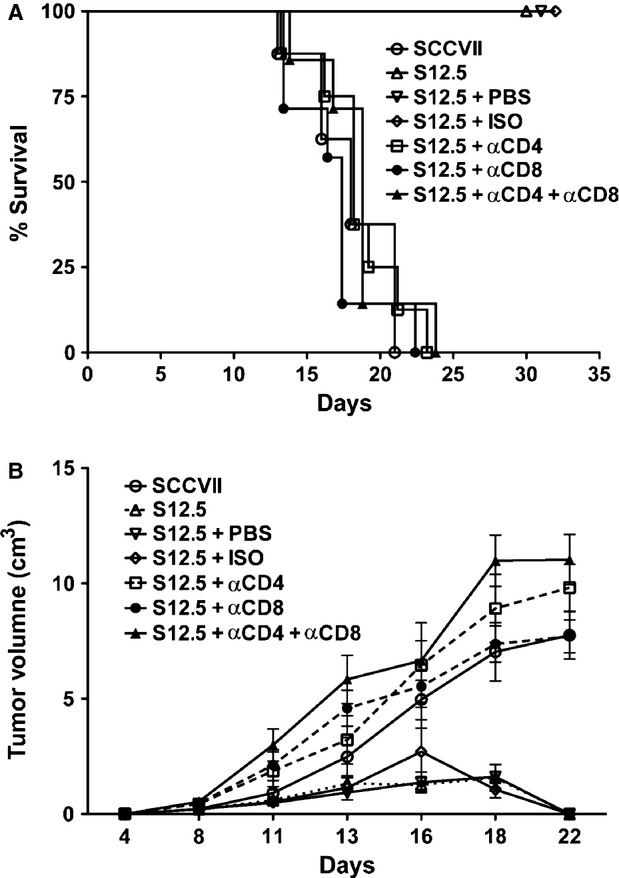
Both CD4^+^ and CD8^+^ cells are required for cell-mediated tumour protection following vaccination with interleukin (IL)-12-producing tumour cells. *A,* C3H/HeJ mice were depleted for CD4^+^ and CD8^+^ cells using specific antibodies. Mice were then challenged with 2x10^5^ IL-12-secreting S12.5 cells s.c. into the right flank and monitored for tumour development. (*P* < 0.05, *n* = 8, log-rank test). *B,* Tumour growth of groups described in *A*.

To determine whether the elimination of the IL-12-producing cells was due to a T cell-dependent anti-tumour immune response, we repeated these experiments using mice in which we selectively depleted CD4^+^ cells and/or CD8^+^ cells (Fig. 3). The depletion of either population eliminated anti-tumour immunity and allowed tumour progression. In these cases, tumour cell growth kinetics and mouse survival times were similar to findings using non-IL-12-producing tumour cells in immune-competent mice.

### IL-12-secreting tumour cells lead to the establishment of immune memory

**Figure 4:**
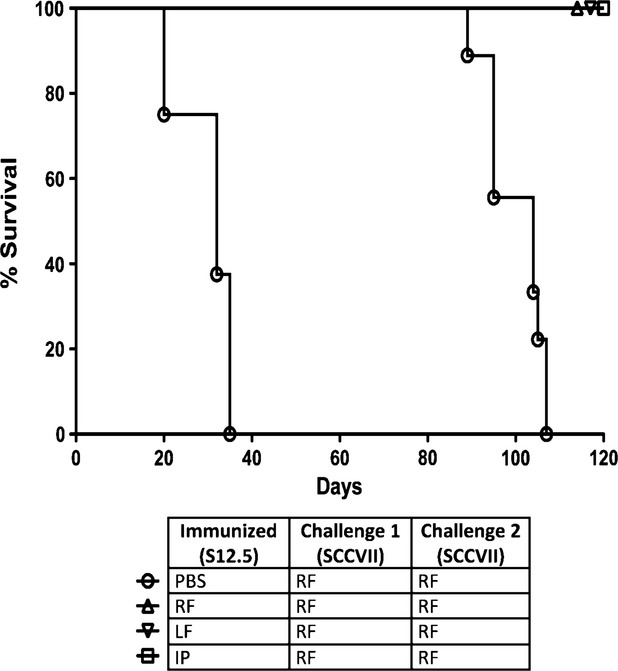
Injection of interleukin (IL)-12-secreting clone S12.5 led to development of long-term immune memory. C3H/HeJ mice were immunized with S12.5 or PBS s.c. at sites indicated (RF: right flank, LF: left flank, IP: intraperitoneal). Fifty days after immunization, mice were challenged with non-IL-12-secreting SCCVII s.c. in the right flank (Challenge 1). Seventy days after challenge 1, mice were again challenged with SCCVII s.c. in the right flank (Challenge 2) with another group of PBS-injected mice acting as controls. All S12.5 immunized mice were protected against subsequent challenge with SCCVII. *P* < 0.05, *n* = 8, log-rank test.

To determine whether the anti-tumour immune response generated by vaccination with IL-12-producing cells was long-lasting, we immunized the mice with either PBS or clone S12.5, and challenged them with parental SCCVII 50 days later. As seen in Figure 4, all PBS-immunized mice developed tumours, whereas all S12.5-immunized mice were protected from tumour development (*P* < 0.05, *n* = 5). In these experiments, protection was observed when mice were initially injected in the left flank, right flank or IP and subsequently challenged by injection in the right flank, suggesting that systemic immunity was achieved. To further validate this, the same mice were challenged again 70 days later, with another group of PBS-injected mice acting as controls, and the same observations were seen (Fig. 4).

### IL-12-secreting tumour cells lead to higher levels of tumour infiltrating lymphocytes (TILs)

**Figure 5:**
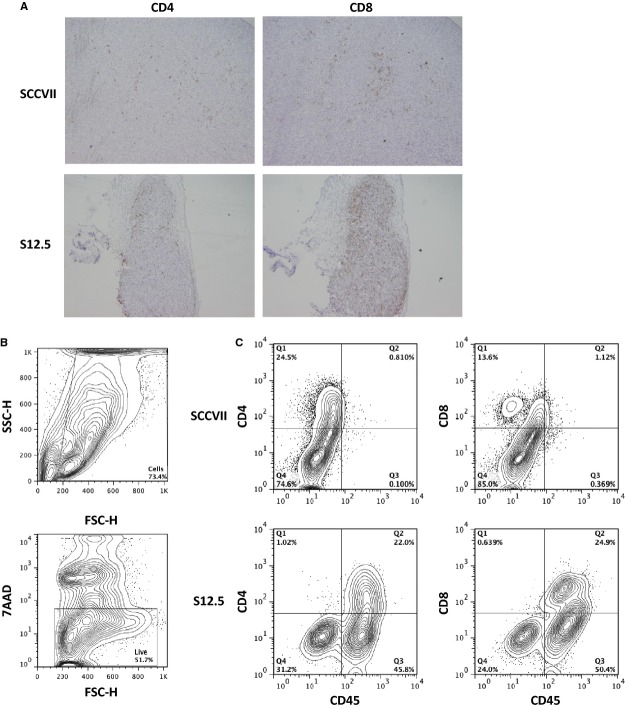
Immunostaining and flow cytometry analysis of tumour isolated from SCCVII or S12.5-injected mice on day 15 post-injection. Experiments were performed three to four times and representative examples presented. (A) Histology sections of tumour isolated from SCCVII (top row) or S12.5 (bottom row) injected mice stained with αCD4 (left column) and αCD8 (right column). All histology sections are shown at 20 ×  magnification. (B) Gating strategy. Debris was gated out using FSC and SSC, 7AAD was then used to distinguish between live and dead cells. (C) Live cells were analysed on their expression of CD45, CD4 and CD8.

We next analysed the TILs in tumours formed by SCCVII and S12.5 cells in immunocompetent mice (Fig. 5). When tumour cross-sections were analysed by immunohistochemistry, S12.5 tumours were found to have a significant increase in CD4^+^ and CD8^+^ cells compared with SCCVII tumours. When the tumours were analysed *via* flow cytometry, tumours formed by S12.5 cells contained larger populations of CD45^+^CD4^+^ cells and CD45^+^CD8^+^ cells (28.8% and 17.3% respectively), compared with tumours formed by SCCVII cells.

**Figure 6:**
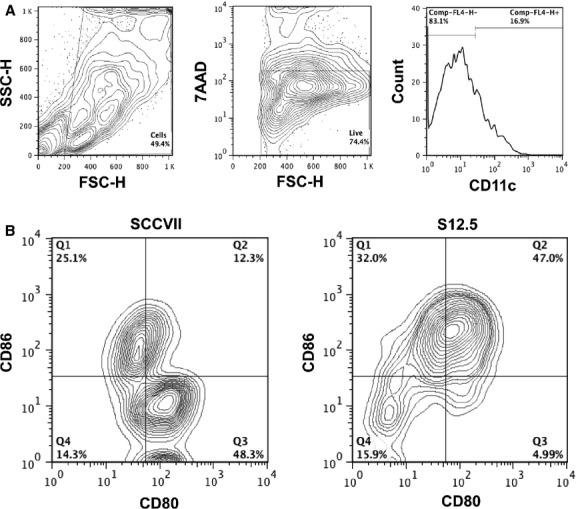
Flow cytometry analysis of dendritic cells (DC) within SCCVII or S12.5 tumours on day 15. Experiments were performed three to four times and representative examples shown. (A) Gating strategy. Debris was gated out using FSC and SSC, 7AAD was then used to gate on the live cells. Within the live cell gate, DCs were gated on using CD11c. (C) Expression of CD80 and CD86 by DC within SCCVII and S12.5 tumours.

In addition, CD80 and CD86 surface expression was used to gauge the activation profile of CD11c^+^ DC found within the tumours. While tumours from mice injected SCCVII cells contained DCs expressing either CD80 or CD86 alone, tumours formed by S12.5-injected mice contained DCs that co-expressed these two co-stimulatory molecules (Fig. 6).

### A small percentage of IL-12-producing cells are able to protect mice from tumour development

**Figure 7:**
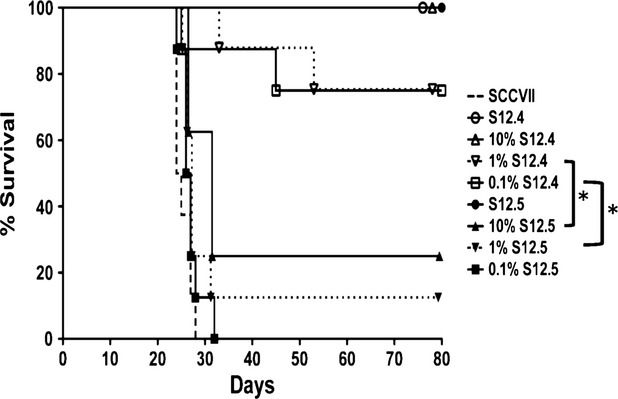
A small percentage of interleukin (IL)-12-secreting cells are sufficient to protect the mice from developing tumours. C3H/HeJ mice were injected s.c. in the right flank a mixture containing IL-12-secreting cells (at concentrations indicated) and non-secreting SCCVII cells. IL-12-secreting clone S12.4 secretes IL-12 at a concentration 10-fold greater than S12.5 (S12.4 secretes 5 ng/ml when plated at 10^6^ cells/ml/4 hrs, whereas S12.5 secretes 0.5 ng/ml). *P* < 0.05, *n* = 8, log-rank test.

As described previously in our murine leukaemia model 11, a key parameter of this cell-based anti-cancer therapy is the amount of IL-12 produced on a per cell basis. To determine if this is also the case in our solid tumour models, cell mixtures were made consisting of IL-12-producing cells and non-transduced SCCVII cells. The two cell lines chosen for this study were S12.4 and S12.5 (5 ng/ml/10^6^ cells/4 hrs and 0.5 ng/ml/10^6^ cells/4 hrs respectively). For these experiments, we chose ratios of non-producing to producing cells of 9:1, 99:1 and 999:1. These mixtures were injected s.c. into the right flank of mice, which were then monitored for tumour development (Fig. 7). As expected, injections of 100% SCCVII cells led to tumour development in all cases, and injection of either of the IL-12-producing cell lines before mixing with non-producing cells resulted in complete protection. As seen in Figure 7, the mixing experiment revealed that both IL-12-producing lines elicit some protection when present as only a fraction of the mixture. The high producing clone, S12.4, when representing as little as 0.1% of the mixture, was able to protect the majority of mice. In contrast, the low producing clone, S12.5, while protecting some mice when present at 10% and 1% of the mixture, was unable to protect mice when present at 0.1%. Direct comparisons were made between 1% S12.4 and 10% S12.5 as well as 0.1% S12.4 and 1% S12.5 because the total *in vitro* production of IL-12 is equal under these circumstances because of a 10-fold difference in the IL-12 production levels of these two clones (*P* < 0.05, *n* = 8).

### Cell-based IL-12 therapy does not lead to elevated systemic levels of IFN- γ or TNF-α

To determine if the introduction of IL-12-producing cells perturbed systemic cytokine levels, we examined blood serum samples taken from mice injected PBS, SCCVII or S12.5 on days 1, 5, 7, 10, 12, 17 and 22 after injection. Over the course of tumour rejection, neither IFN-γ nor TNF-α was detectable (data not shown).

### Similar protection is observed in other murine solid tumour models

**Figure 8:**
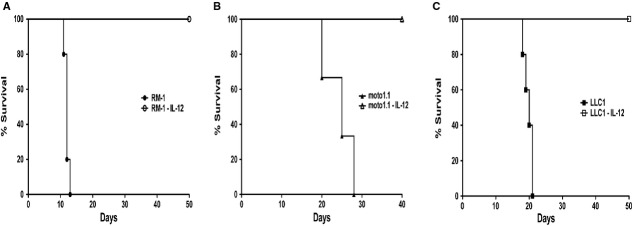
Analogous outcome is seen with other solid tumour models. (A) C57B/L/6J mice injected s.c. on the right flank interleukin (IL)-12-secreting or non-secreting prostate cancer RM-1 cells (2 × 10^4^ cells/200 μl/mouse). (B) FVB/NJ mice injected s.c. on the right flank IL-12-secreting or non-secreting osteosarcoma moto1.1 cells (10^6^ cells/50 μl/mouse). (C) C57BL/6J mice injected s.c. on the right flank IL-12-secreting or non-secreting lung carcinoma LLC1 cells (1 × 10^6^ cells/200 μl/mouse).

To determine if our observations using the SSCVII tumour model could be extended to other solid tumours, we repeated these experiments using the Lewis lung carcinoma cell line, LLC1, prostate cancer cell line, RM-1, and the osteosarcoma line, moto1.1. After transducing the cell lines with our murine IL-12 lentivector, clones were isolated that produce IL-12. These cells were then injected into C57BL/6J (for RM-1 and LLC1) and FVB/NJ (for moto1.1) mice, and tumour development was monitored (Fig. 8). All of the mice that were injected IL-12-producing cells were protected from tumour development, whereas mice injected non-IL-12-producing cells developed tumours.

## Discussion

Interleukin-12 was discovered more than 20 years ago 7–8 and has proven to be a key mediator determining the balance between immune activation and immune tolerance. It plays important roles in priming Th1 responses. In response to microbial pathogens, the IL-12 produced by cells such as macrophages and DC is a key cytokine in pushing Th1 differentiation, bridging the innate and adaptive immune responses 7–8. Lastly, Voest *et al*. demonstrated anti-angiogenic properties of IL-12 22.

The properties noted above mark IL-12 as a potential agent used for therapeutic intervention in diseases, such as cancer, characterized by an insufficient immune response. In fact, a number of clinical trials have been carried out using IL-12 23. Systemic therapy has been tried in renal cell carcinoma, melanoma and colon cancer 24. Unfortunately, unacceptable levels of toxicity were observed using this systemic IL-12 delivery. Therefore, alternative delivery models have been under active investigation.

In this study, IL-12 was delivered *via* the tumour cells themselves, which we engineered to express IL-12 using a lentiviral vector system. The efficacy of this method has already been reported in mouse solid tumour models 25–26. In those models, it was demonstrated that tumour cells could be transduced with adenoviral or other retroviral systems to produce IL-12. The injection of these cells led to a decreased growth rate of the tumour, correlating with better survival. Furthermore, tumour cells that produce IL-12 led to long-lasting antitumour responses.

On the basis of these promising results, we undertook additional experiments using IL-12. First, we established a vector system based on the use of recombinant lentivirus. Lentiviruses efficiently incorporate their DNA into the host and maintain IL-12 production even as the cells divide, thereby allowing for propagation of the transduced population. We demonstrated in a murine leukaemia model that the lentiviral vector can efficiently transduce these cells and maintain their IL-12 expression for extended periods 11. Furthermore, IL-12-producing cells are able to protect the mice from leukaemia development. Importantly, we found that the amount of IL-12 produced per cell is a key determinant in eliciting a curative immune response, while also demonstrating that only a very low percentage of IL-12-producing cells are required. Given that leukaemias represent only a minority of human cancers, it was critical to determine if similar results could be found in other tumour models. In this present study, we expand these observations to solid tumours.

For the evaluation of the mIL-12 cell-based therapy in solid tumours, we first selected a well-characterized cell line, SCCVII, which forms tumours in immune-competent mice. Using the IL-12-producing vector previously described 11, the cells were transduced with LV/mIL-12, clones obtained by limiting dilution, and cell lines established that secrete different levels of IL-12. The expression of IL-12 by SCCVII cells prevented tumour establishment in 100% of the mice when injected s.c. (Fig. 2A). We previously demonstrated that rejection is IL-12-mediated, rather than a function of the vector system, as leukaemia clones that were transduced but did not express sufficient levels of IL-12 were not protective 11. In the present solid tumour system, we have yet to isolate a clone that expresses so little IL-12 that it is not protective when injected alone, unmixed with the parent line. To control for heterogenous tumourigenicity in the parent population, untransduced SSCVII cells were also cloned and each clone formed tumours upon injection. In the case of the lowest IL-12-producing clone, S12.5 (IL-12 production at 0.5 ng/ml when cells were at a density of 1 × 10^6^ cells/ml for 4 hrs), a visible tumour appeared that reached a maximum size at 2 weeks after which it regressed, presumably as a result of immune activation. The ability of these cells to develop a tumour and then be rejected provided us with a functional model in which we could further study the mechanisms of action within the tumour microenvironment.

Two pieces of evidence presented in this study support the concept that SCCVII cells expressing IL-12 are capable of inducing active immunity. First, when either CD4^+^ or CD8^+^ cell populations are eliminated, the IL-12-producing cells lose their ability to protect the mice from tumour development. Consistent with this, we observed elevated numbers of both CD4^+^ and CD8^+^ cells in tumours initially formed by S12.5 cells as compared with tumours that developed in mice injected parental SCCVII cells (Fig. 5). Secondly, 100% of mice immunized with IL-12-producing cells were protected from subsequent challenge with non-IL-12-producing cells, while control animals developed tumours 100% of the time. This indicates that the initial immunization was able to establish immune memory against SCCVII. These experiments do not rule out a role for other mechanisms of tumour clearance and, indeed IL-12 is known to induce robust multifaceted immune responses. However, T cells play a dominant role in the response as evidenced by the fact that the survival curves for mice depleted of either T-cell population, or both together, are not significantly different from that for mice injected with the parental line.

Of most relevance to the extension of these observations to human studies is that the level of IL-12 production per cell is critical. When comparing mice injected a mixture containing 10% of low IL-12-producing cells (S12.5) with mice injected a mixture containing 1% of high IL-12-producing cells (S12.4), tumours containing low numbers of high IL-12-producing cells were eliminated, whereas tumours containing 10-fold higher numbers of low IL-12 producers were not. This result suggests that when tumour cells encounter the immune system, a threshold of IL-12 is required to achieve activation and anti-tumour immunity. Once activated, the immune system is able to eliminate both IL-12-producing and non-IL-12-producing cells. Our findings suggest that human clinical trials must focus not only on the percentage of cells transduced but also on the amount of IL-12 produced by single cells.

Clinical trials involving systemic injections of recombinant IL-12 for treating melanoma led to toxicity that was attributed to high IFN-γ levels in the serum. This prompted us to look at various inflammatory cytokines in mice injected IL-12-secreting tumour cells. We selected S12.5 for these studies because this allowed us to compare cytokine levels during both the progression and regression phases of tumour growth. In these experiments, we failed to find consistent increases in these cytokines when comparing S12.5-injected mice with SCCVII- or PBS-injected mice. Of interest, the amount of IFN-γ in the serum was below the level of detection, suggesting that IL-12 delivered by the tumour cells themselves can induce immunity without causing what could be a toxic rise in IFN-γ.

Our results using a leukaemia line 11 as well as cell lines representing osteosarcoma, Lewis lung carcinoma, squamous cell carcinoma, and prostate carcinoma suggest that delivery of IL-12 by cancer cells is an effective and generalizable route to immune activation. We conclude that IL-12, if delivered by tumour cells themselves in sufficient amounts, can activate anti-tumour immunity. Our results suggest that the use of IL-12 as an immunotherapeutic agent should be re-examined in clinical settings using autologous neoplastic cells engineered to express IL-12 with attention given not only to obtaining a high yield of IL-12-secreting tumour cells, but also to the level of IL-12 produced by individual cells.
